# Trastuzumab emtansine is active on HER-2 overexpressing NSCLC cell lines and overcomes gefitinib resistance

**DOI:** 10.1186/1476-4598-13-143

**Published:** 2014-06-05

**Authors:** Daniele Cretella, Francesca Saccani, Federico Quaini, Caterina Frati, Costanza Lagrasta, Mara Bonelli, Cristina Caffarra, Andrea Cavazzoni, Claudia Fumarola, Maricla Galetti, Silvia La Monica, Luca Ampollini, Marcello Tiseo, Andrea Ardizzoni, Pier Giorgio Petronini, Roberta R Alfieri

**Affiliations:** 1 Department of Clinical and Experimental Medicine, University of Parma, Via Gramsci 14, Parma, 43126, Italy; 2 Department of Biomedical, Biotechnological and Translational Sciences, University of Parma, Parma, Italy; 3 Italian Workers’ Compensation Authority (INAIL) Research Centre at the University of Parma, Parma, Italy; 4 Thoracic Surgery, Department of Surgical Science, University Hospital of Parma, Parma, Italy; 5 Division of Medical Oncology, University Hospital of Parma, Parma, Italy

**Keywords:** Lung cancer, HER-2, Trastuzumab, T-DM1

## Abstract

**Background:**

HER-2 represents a relatively new therapeutic target for non small cell lung cancer (NSCLC) patients. The incidence for reported HER-2 overexpression/amplification/mutations ranges from 2 to 20% in NSCLC. Moreover, HER-2 amplification is a potential mechanism of resistance to tyrosine kinase inhibitors of the epidermal growth factor receptor (EGFR-TKI) (about 10% of cases). T-DM1, trastuzumab emtansine is an antibody-drug conjugate composed by the monoclonal antibody trastuzumab and the microtubule polymerization inhibitor DM1. The activity of T-DM1 has been studied in breast cancer but the role of T-DM1 in lung cancer remains unexplored.

**Methods:**

Antiproliferative and proapoptotic effects of T-DM1 have been investigated in different NSCLC cell lines by MTT, crystal violet staining, morphological study and Western blotting. HER-2 expression and cell cycle were evaluated by flow cytometry and Western blotting. Antibody dependent cell cytotoxicity (ADCC) was measured with a CytoTox assay. Xenografted mice model has been generated using a NSCLC cell line to evaluate the effect of T-DM1 on tumor growth. Moreover, a morphometric and immunohistochemical analysis of tumor xenografts was conducted.

**Results:**

In this study we investigated the effect of T-DM1 in a panel of NSCLC cell lines with different HER-2 expression levels, in H1781 cell line carrying HER-2 mutation and in gefitinib resistant HER-2 overexpressing PC9/HER2cl1 cell clone. T-DM1 efficiently inhibited proliferation with arrest in G2-M phase and induced cell death by apoptosis in cells with a significant level of surface expression of HER-2. Antibody-dependent cytotoxicity assay documented that T-DM1 maintained the same activity of trastuzumab. Our data also suggest that targeting HER-2 with T-DM1 potentially overcomes gefitinib resistance. In addition a correlation between cell density/tumor size with both HER-2 expression and T-DM1 activity was established in vitro and in an in vivo xenograft model.

**Conclusions:**

Our results indicate that targeting HER-2 with T-DM1 may offer a new therapeutic approach in HER-2 over-expressing lung cancers including those resistant to EGFR TKIs.

## Background

A number of molecular aberrations have been identified in non small cell lung cancer (NSCLC), including EGFR, BRAF, HER2 mutations, EML4-ALK, ROS1 and RET rearrangements in adenocarcinoma; FGFR mutations/amplifications, DDR2 or PIK3CA mutations in squamous cell carcinoma [1]. Conflicting results have demonstrated marginal benefit of targeted molecules in unselected populations of patients with advanced NSCLC. However, some targeted agents have been approved in different line settings for the treatment of specific subgroups of patients [2-5]. In particular, the epidermal growth factor receptor (EGFR) has been successfully targeted in NSCLC patients harbouring activating-EGFR mutations by small molecules inhibiting the tyrosine kinase domain (gefitinib, erlotinib and afatinib) [2-4]. Moreover, crizotinib has been approved by US Food and Drug Administration (FDA) and European Medicines Agency (EMA) for the treatment of advanced or metastatic ALK positive NSCLC patients [5,6].

The acquisition of resistance to tyrosine kinase inhibitors (TKIs) in clinical oncology is a well documented phenomenon that applies to several types of cancers. Almost all NSCLC patients with activating EGFR mutations treated with EGFR-TKI, after an initial response, experience disease progression within 10 to 14 months from the beginning of the therapy [7]. A commonly described mechanism of drug resistance involves additional genetic alterations within the EGFR itself, the most frequent being the T790M mutation accounting for approximately 50% of cases of acquired resistance [8]. An additional well documented mechanism is MET amplification initially reported in 15-20% of resistant patients [9] but recently reduced to 3-5% [10,11]. Several other pathways have been associated with resistance to EGFR TKI including histologically documented transformation to small phenotype, PIK3CA mutation and epithelial to mesenchymal transition [12].

HER-2 represents a relatively new therapeutic target for NSCLC. The potential clinical relevance of HER-2 expression in NSCLC is currently under evaluation [13], however, the recent role of HER-2 amplification in the acquisition of resistance to TKI, reported in 12-13% of patients [11,14], may render HER-2 a potential target not only in breast cancer but also in NSCLC.

T-DM1, trastuzumab emtansine, is an antibody-drug conjugate composed by the microtubule polymerization inhibitor DM1 (derivative of maytansine) linked with a stable thioether linker to trastuzumab, a monoclonal antibody that targets HER-2 receptor [15]. After binding to HER-2 receptors, the complex undergoes internalization and lysosomal degradation with the release of DM1 active catabolites that bind to tubulin and suppress microtubule dynamics [16].

The activity of T-DM1 has been extensively studied in several human breast cancer cell lines [15,17,18] showing a superior activity compared to trastuzumab in HER-2 overexpressing cells. T-DM1 has been recently approved for the treatment of HER-2-positive metastatic breast cancer patients previously treated with trastuzumab and taxane [19].

The aim of the present study was to test whether T-DM1 activity is affected by HER-2 expression/mutation status and may overcome EGFR-TKI resistance in NSCLC cell lines. To this purpose we evaluated the effect of T-DM1 in a panel of NSCLC cell lines with different HER-2 expression levels, in H1781 cell line carrying HER-2 mutation [20], and in gefitinib resistant HER-2 overexpressing PC9/HER2cl1 cell clone [14]. Moreover, we explored the correlation between cell density/tumor size, HER-2 expression and T-DM1 activity in vitro and in an in vivo xenograft model.

## Results

### HER-2 expression level in human non-small-cell lung cancer cell lines

**Figure 1:**
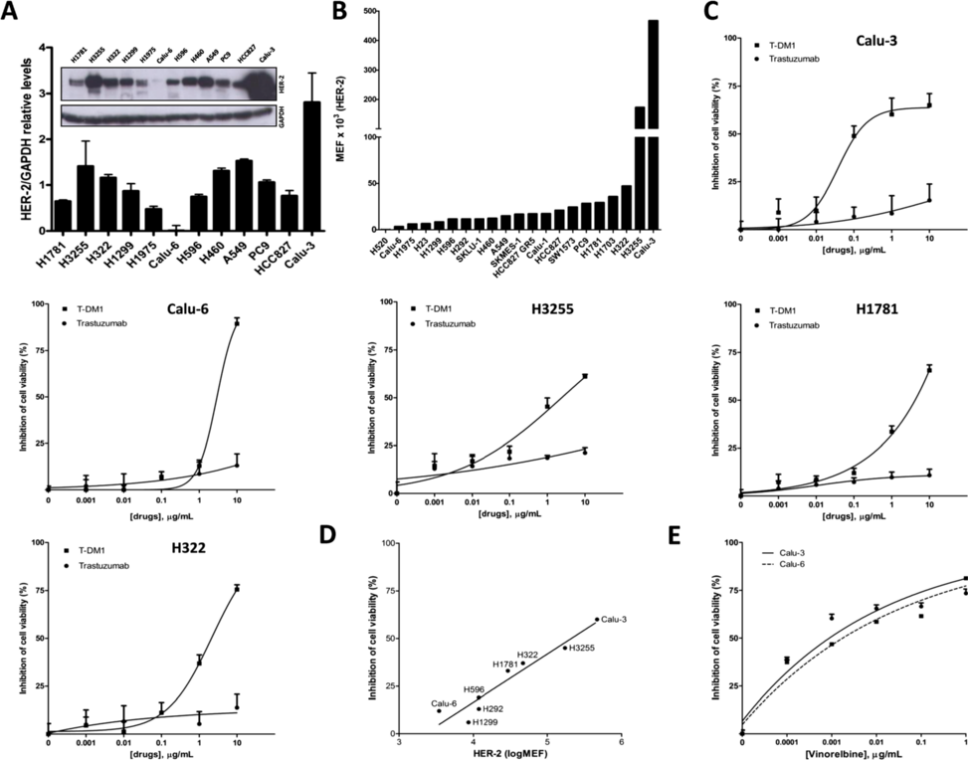
**HER-2 levels and effects on cell viability of T-DM1, trastuzumab and vinorelbine in NSCLC cell lines. (A)** Densitometric quantification of total HER-2 protein level, detected by Western blotting, was calculated using Quantity One software. Three different Western blot experiments were performed on total cell lysate of the indicated NSCLC cell lines. A representative Western blot analysis is reported as inset. **(B)** HER-2 protein levels on cell surface was quantified by flow-cytometry and expressed as molecular equivalent of fluorochrome (MEF) as described in Methods section. **(C)** Calu-3, Calu-6, H3255, H1781 and H322 cells were exposed to increasing concentrations (0.001, 0.01, 0.1, 1 and 10 μg/ml) for 72 h and then cell viability was assessed by MTT assay. **(D)** Percent of inhibition of cell viability induced by T-DM1 at 1 μg/ml as a function of HER-2 level **(E)** Calu-3 and Calu-6 cell viability inhibition curves after treatment with increasing vinorelbine concentrations. Data are expressed as mean + SD of three different experiments.

The total level of HER-2 protein was detected by immunoblotting on cell lysates in a panel of NSCLC cell lines (H1781, H3255, H322, H1299, H1975, Calu-6, H596, H460, A549, PC9, HCC827 and Calu-3). As shown in Figure 1A, HER-2 expression varied widely among the analyzed cell lines, ranging from barely detectable levels in Calu-6 to high levels in Calu-3. The latter was an expected finding, due to known amplification of HER-2 in Calu-3 cell line [21]. Considering that trastuzumab and T-DM1 have a common targeted receptor on the cell surface, we quantified HER-2 expression levels on the plasma membrane by flow cytometry. Indeed, the ability of the antibody to interact with its target is strictly related to the presence of the receptor on the cell surface. As reported in Figure 1B, Calu-3 and H3255 cells displayed the highest levels of HER-2 at the plasma membrane. The total level of HER-2 in H3255 was similar to that observed in other cell lines such as H460 and A549 (Figure 1A) indicating that the total level of proteins detected on cell lysate is not a good predictor of HER-2 level on plasma membrane.

### Effect of T-DM1 and trastuzumab treatment on cell viability of NSCLC cell lines with different HER-2 expression

Based on the previous analysis, the effect of T-DM1 and trastuzumab on cell viability was focused on NSCLC cell lines expressing different cell surface levels of HER-2: Calu-3 (very high), H3255 (high) and Calu-6 (low). T-DM1 showed strong anti-proliferative effect in HER-2 over-expressing Calu-3 cells (IC_50_ = 0.40 ± 0.08 μg/ml, Figure 1C), whereas no effects were detected on Calu-6 cell line, with low levels of HER-2 on the plasma membrane. The inhibition observed at 10 μg/ml was related to a non-specific toxic effect of T-DM1, as previously reported in MCF-7 HER-2 negative breast cancer cell line [22]. Intermediate results were seen on H3255 cells. Trastuzumab, administered at the same dosages of T-DM1, did not show notable effect on cell growth in any of the cell lines tested. H1781 cell line, harbouring mutated HER-2 (G776insV_G/C), was also included in this study in order to investigate whether this mutation influenced the anti-proliferative effect of T-DM1. As shown in Figure 1C, T-DM1 at 1 μg/ml induced about 35% inhibition of cell viability. A similar inhibition profile was observed in T-DM1 (1 μg/ml) treated H322 cells, displaying comparable levels of HER-2 on cell surface (Figure 1B), suggesting that H1781 sensitivity to the drug is not affected by the mutated receptor. We then analyzed the percentage of inhibition of cell viability induced by 1 μg/ml T-DM1 on eight different cell lines, as a function of HER-2 level on plasma membrane and, as shown in Figure 1D, we confirmed that sensitivity to T-DM1 is strictly correlated to HER-2 expression on the cell surface.To exclude the hypothesis that different effects of T-DM1 could be ascribed to dissimilar sensitivity to the microtubule polymerization inhibitor DM1, we treated two cell lines harbouring high and low levels of HER-2 on the plasma membrane, respectively Calu-3 and Calu-6, with increasing concentration of Vinorelbine, an anti-mitotic drug, which acts by a similar mechanism of action of the maytansinoid DM-1. Vinorelbine inhibited viability of Calu-3 and Calu-6 cells in a comparable manner (Figure 1E).

### Effect of T-DM1 and trastuzumab treatment on cell cycle distribution, signal transduction, cell death and antibody dependent cell cytotoxicity (ADCC)

**Figure 2:**
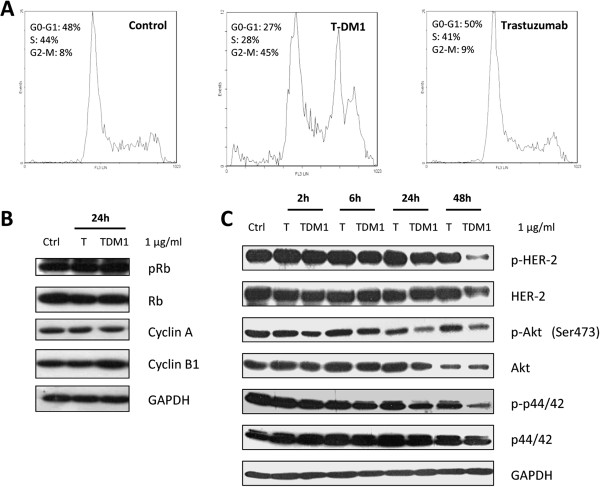
**Influence of trastuzumab and T-DM1 on cell cycle phase distribution and cell signalling. (A)** Calu-3 cells were cultured in the absence of drugs or treated either with T-DM1 or trastuzumab (1 μg/ml). After 24 h cells were stained with propidium iodide an cell-cycle-phase distribution was determined by flow cytometry analysis. Cell-cycle distributions were analyzed as described in Methods section and data were expressed as percentage of distribution in each cell-cycle phase. Immunoblot analysis on protein involved in cell cycle regulation **(B)** or signalling pathways **(C)** were conducted on cell lysates obtained after treatment with trastuzumab or T-DM1 (1 μg/ml) for the indicated period of time.

**Figure 3:**
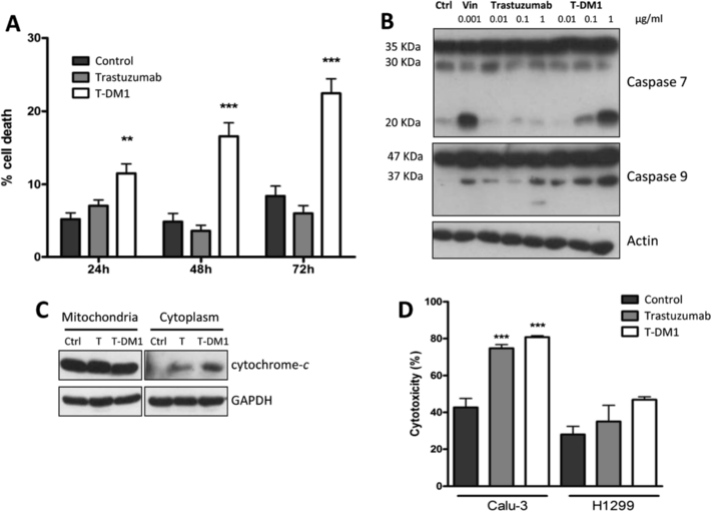
**Effect of trastuzumab and T-DM1 on cell death and antibody dependent cell cytotoxicity. (A)** Dead cells were counted after 24, 48 and 72 h of exposure to trastuzumab or T-DM1 (1 μg/ml) and the percentage of dead cells was calculated. (**p < 0.01, ***p < 0.001 versus control, one-way ANOVA followed by Tukey’s post-test). **(B)** Caspases 7 and 9 activation were detected by immunoblotting on cell lysates obtained after 48 h of Calu-3 exposure to increasing concentration of trastuzumab or T-DM1. Vinorelbine 0.001 μg/ml was used as positive control. **(C)** Cytochrome *c* was detected in the cytoplasm by immunoblotting after 48 h of treatment with T-DM1 1 μg/ml as described in Methods section. **(D)** Trastuzumab (1 μg/ml) or T-DM1 (1 μg/ml) were added to Calu-3 and H1299 cells seeded with 100 U/ml IL-2 activated-NK cells, at the ratio of 1:50. After 4 h lactate dehydrogenase release was quantified as described in Methods section and data expressed as percentage of cytotoxicity. The results are from representative experiments. The experiment, repeated three times, yielded similar results (***P < 0.001, one-way ANOVA followed by Tukey’s post-test).

To determine the effect of T-DM1 on cell cycle, Calu-3 treated with 1 μg/ml T-DM1 or Trastuzumab for 24 h were analyzed by flow cytometry. As shown in Figure 2A, T-DM1 caused an increase in the proportion of cells in G2-M phase with a decrease in G1 and S phases, whereas no alterations on cell cycle distribution were detected in cells treated with trastuzumab. The arrest of Calu-3 in G2-M phase of the cell cycle, as a result of T-DM1 exposure, was also supported by increased levels of Cyclin B1 as measured by western blot analysis (Figure 2B) whereas pRb and Cyclin A were unchanged.We then tested the effect of T-DM1 and trastuzumab on phosphorylation status of HER-2, AKT and p42-44 MAPK in Calu-3 cell line. Differently of trastuzumab, T-DM1 significantly inhibited the phosphorylation of AKT and p42-44 after 24 h of treatment (Figure 2C) with a decrease in HER-2 total level and phosphorylation at 48 h.T-DM1 (1 μg/ml) exerted a significant cytotoxic effect already after 24 h of treatment, with appearance of floating dead cells (Figure 3A). By contrast trastuzumab did not modify cell proliferation nor induced cell death up to 72 h of treatment.

As shown in Figure 3B, 48 h exposure of Calu-3 cells to T-DM1 at 0.1 and 1 μg/ml induced the activation of caspases-7 and −9 and the release of cytochrome-*c* into the cytoplasm (Figure 3C) indicating that the intrinsic pathway is involved in T-DM1-triggered apoptotic cell death. Vinorelbin was used as internal control. A lower activation of caspases and a weak release of cytochrome-*c* was also induced by trastuzumab treatment even if no significant cell death was observed (Figure 3A).

Since antibody-dependent cell-mediated cytotoxicity (ADCC) is one of the main mechanisms of action of specific mAbs directed to ErbB family members in vivo [23], we examined whether the capability to activate natural killer (NK)-mediated ADCC is preserved by T-DM1. As shown in Figure 3D, T-DM1-dependent cytotoxicity in the presence of IL-2 activated NK cells was similar to trastuzumab-dependent cytotoxicity in Calu-3 overexpressing HER-2. In the low HER-2 expressing H1299 cells, neither T-DM1 nor trastuzumab significantly induced mAb-dependent cytotoxicity.

### Effect of T-DM1 on EGFR-mutant PC9 cell line resistant to gefitinib for HER-2 overexpression

**Figure 4:**
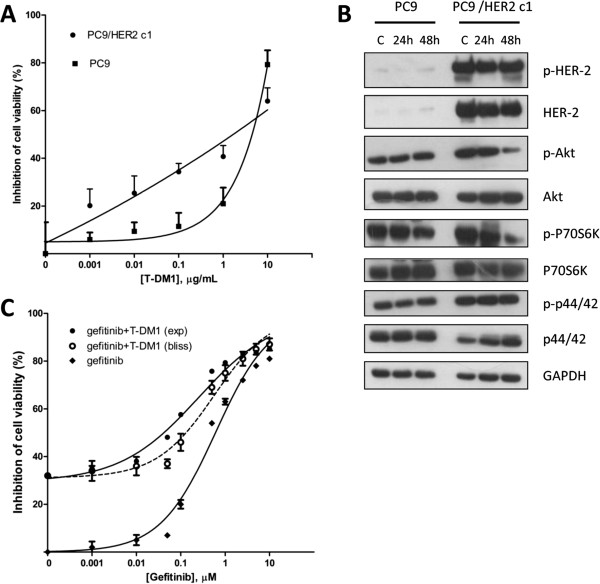
**Effect of T-DM1 on EGFR-mutant PC9 cell line become resistant to gefitinib for HER-2 overexpression. (A)** PC9 and PC9/HER2 c1 cells were exposed to increasing concentrations of T-DM1 for 72 h and then cell viability was assessed by MTT assay. Data are expressed as mean + SD of three different experiments. **(B)** Immunoblot analysis of proteins of signalling transduction pathways were conducted on cell lysates obtained after treatment with T-DM1 (1 μg/ml) for 24 or 48 h. **(C)** Curves of growth inhibitory effects of gefitinib and combined treatment gefitinib plus T-DM1 versus theoretical Bliss additivity curve are reported. Cells were treated with the drugs for 72 h and then cell number was assessed by MTT assy. Data are expressed as percent inhibition of cell proliferation versus control cells. The experiments, repeated three times, yielded similar results.

As previously reported [14] and independently confirmed by our laboratory, the clone PC9/HER2c1 (a generous gift from Dr. William Pao), obtained by stably transfection of PC9 cells with HER-2 expression vector, is more resistant to gefitinib than parental cells. HER-2 expression on plasma membrane was 10 time higher in the clone compared to the parental cell line (data not shown).Based on these results we tested the effect of T-DM1 on PC9/HER2c1 and in the parental PC9 cells. As shown in Figure 4A, HER-2 overexpression significantly enhanced the efficacy of T-DM1 with 40% inhibition of cell viability at 1 μg/ml in the PC9/HER2c1 clone. With respect to PC9 cells, the clone showed a marked increase in AKT, p70S6K and p42-44 activation. After 48 h of treatment with T-DM1 a reduction in AKT and p70S6K phosphorylation was observed (Figure 4B) suggesting that T-DM1 might improve gefitinib treatment. In Figure 4C the dose–response curves of gefitinib in the presence of a fixed concentration of T-DM1 (0.1 μg/ml) are shown. Comparing the experimental combination points with that expected by the Bliss criterion, an additive effect was observed. In fact, no significant differences between experimental and theoretical points were observed.

### In vivo activity of T-DM1 is dependent on tumor size and HER-2 expression

**Figure 5:**
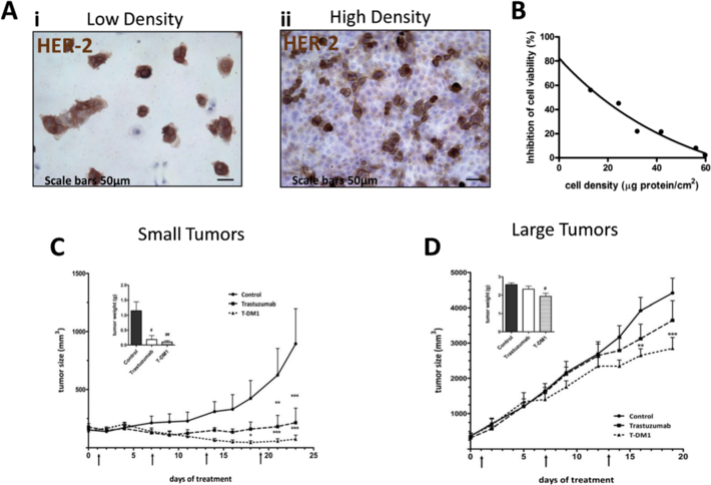
**Cell density in vitro and tumor size in vivo influenced HER-2 expression and efficacy of T-DM1. (A)** Calu-3 cells were plated at low (10^4^cells/cm^2^) (i) and high (8x10^4^cells/cm^2^) (ii) density and after 24 h membrane HER-2 protein expression was evaluated by immunohistochemistry. **(B)** Calu-3 cells were plated at different density and exposed for 72 h to T-DM1 1 μg/ml and then cell number was assessed using crystal violet staining as described in Methods section. Percent inhibition of cell proliferation versus control cells was plotted as function of cell density. The experiments, repeated three times, yielded similar results. 4 × 10^6^**(C)** or 8 × 10^6^**(D)** Calu-3 cells were subcutaneously implanted on BALB/c-Nude mice. At the beginning of the treatments average tumor volumes were 161 ± 15 mm^3^ and 370 ± 50 mm^3^ respectively. In both settings vehicle, trastuzumab (15 mg/Kg i.p.) or T-DM1 (15 mg/Kg i.v.) were administered every six days as pointed (arrows). Tumor sizes were measured three times per week and data expressed as volume + SEM (n = 6 mice per group). (**p < 0.01, ***p < 0.001 vs control; two-way ANOVA followed by Bonferroni’s post-test). After sacrifice tumors were excised and weighted (# p < 0.05 ##p < 0.01; one-way Anova followed by Tukey’s post-test).

It has been reported that cell density can influence the expression of EGFR in breast cancer [24] and in pancreatic cancer cell lines [25] and that surface expression of HER-2 is regulated post-transcriptionally in mammary epithelial cells by the culture cell density [26]. We investigated the dependence of HER-2 membrane protein expression on cell density as well as the effect of T-DM1 on cells seeded at different densities. Confluent Calu-3 cells exhibited a significant decrease of HER-2 at cell surface level detected by immunohistochemistry (Figure 5A ii), as compared to cells seeded at low density (i). At low density, more than 80% of cells showed a strong surface expression of the receptor whereas in almost confluent Calu-3 cultures a significant downregulation of HER-2 was observed (nearly 35%). Consequently, as reported in Figure 5B, the inhibition of cell viability induced by T-DM1 at 1 μg/ml was markedly decreased with increasing cell density.

We performed an in vivo experiment, aimed to determine whether T-DM1 efficacy might be affected by tumor size and structural organization. Tumors were clearly visible in all mice inoculated with 4 × 10^6^ or 8 × 10^6^ Calu-3 with a mean volume of 161 ± 15 mm^3^ (Figure 5C) and 370 ± 50 mm^3^ (Figure 5D) respectively. Trastuzumab (15 mg/Kg intraperitoneal) or T-DM1 (15 mg/Kg intravenously) were given every six days. T-DM1 or trastuzumab treatments in animals carrying tumors of small size were able to strongly inhibit tumor growth compared to vehicle treated mice (Figure 5C). Treatment with T-DM1 not only inhibited tumor growth, but a reduction of tumor dimension was observed in five out of six mice.On the other hand, when treatments were performed on larger tumors, only T-DM1 was able to significantly reduce tumor growth compared to control group, whereas no significant effect was seen with trastuzumab (Figure 5D). We did observe neither rapid tumor shrinkage nor long term complete response in T-DM1 treated animals. T-DM1 in vivo efficacy was confirmed by the reduction in weight of tumors excised at sacrifice, compared to control (Inset to Figure 5C and D).

**Figure 6:**
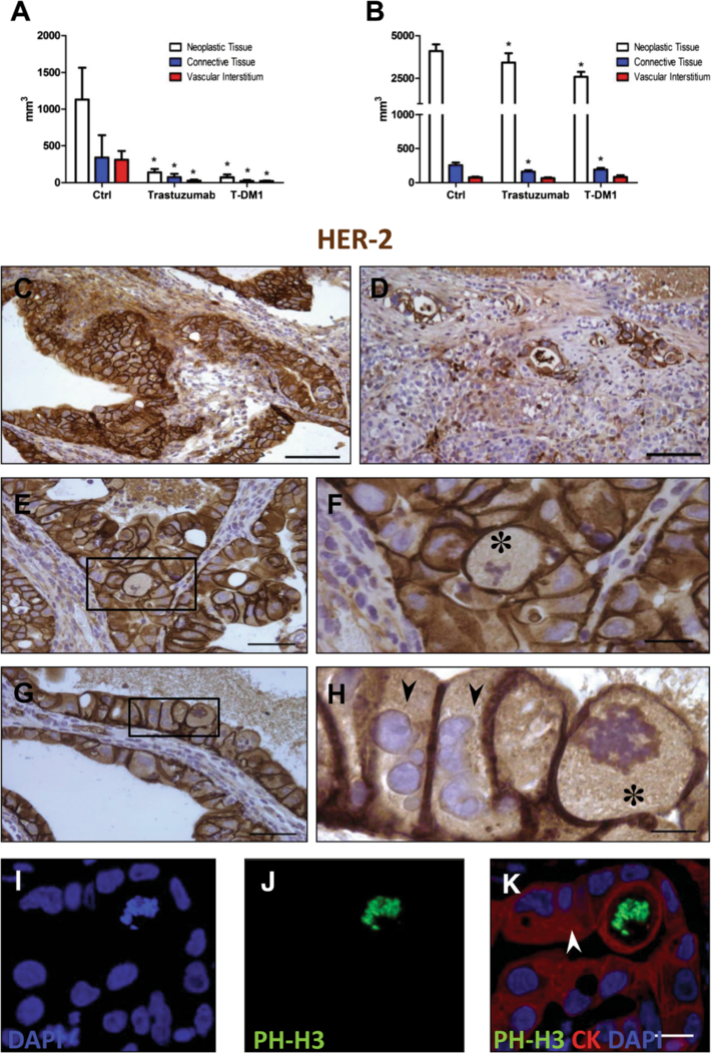
**Neoplastic tissue composition and HER-2 expression in small and large tumors.** Quantification of tissue composition in small **(A)** and large **(B)** tumour xenografts in untreated (CTRL) and Trastuzumab or T-DM1 treated mice (*p < 0.05, vs control) was evaluated as described in Methods Section. C-H: Immunoperoxidase staining of xenografts by anti-HER-2 antibodies. The sharp difference in HER-2 expression (brownish) by neoplastic cells composing small **(C)** and large **(D)** tumours is apparent. **E** and **G** illustrate sections of small T-DM1 treated tumour xenografts in which black rectangles include a microscopic field shown at higher magnification in **F** and **H**, respectively, to document giant mitotic figures (✽) on HER-2 labeled cells. Arrows indicate polynucleated HER-2 positive neoplastic cells. The lower panels show the specific immunofluorescent labeling of metaphase chromosomes **(I)** by phospho-Histone H3 (PH-H3, green, **J**) on a large cytokeratin (CK, red, **K**) positive cell. Arrow points to a giant polynucleated neoplastic cell. Scale bars: C, D = 100 μm; E, G = 50 μm; F, I, L, M = 20 μm and H = 10 μm.

To define at tissue level the response of small and large tumors to T-DM1, a morphometric analysis of neoplastic tissue composition was performed. To this purpose, the fractional volume occupied by PanCK^pos^ cells was assessed (Figure 6A,B). Quantitatively, compared to control in small tumors a 67% and 73% reduction of neoplastic epithelial cells within the nodules was observed with trastuzumab and T-DM1, respectively (A). In large tumors, the amount of neoplastic tissue was reduced by 16% and 37%, respectively, with trastuzumab and T-DM1 compared to control (B). Importantly, both drugs significantly decreased mitotic index in small and large tumors, however, the antiproliferative activity of T-DM1 was superior to that of trastuzumab (data not shown). To gain insights on the mechanism underlying these effects of T-DM1, we sought to determine whether the results obtained in vitro on the different sensitivity to T-DM1 according to cell density and HER-2 expression had an in vivo counterpart. To this hand, sections of small and large tumors were immunostained with HER-2 antibodies (Figure 6C,D). Again, differences in the surface expression of the HER-2 was striking, as neoplastic cells composing small tumors (C) showed higher extent and intensity of HER 2 immunolabelling than large tumors (D). Thus, our data clearly document that the anti-tumor potency of T-DM1 is strictly dependent on HER-2 expression, which in turn is intrinsically modulated among neoplastic cells and their structural organization. Characteristic and atypical mitotic figures and giant multinucleated cells were detected both morphologically on HER-2^pos^ cells (Figure 6E,F,G,H) and by the nuclear labelling of PH-H3 in PanCK expressing cells (Figure 6I,J,K) both in small (Figure 6E,F,G,H) and large tumors (not shown).

## Discussion

One of the major findings of our study is that targeting HER-2 with Trastuzumab-DM1, an antibody-drug conjugate developed to improve the treatment of HER-2 positive breast cancer, may offer a new therapeutic approach in lung cancers expressing HER-2 even when resistant to EGFR TKIs. We also demonstrated that HER-2 is highly expressed in low density NSCLC cells in vitro and in small tumors in vivo by which mechanism T-DM1 exerts a stronger efficacy.

The involvement of HER-2 in lung carcinogenesis has been known for many years but clinical research was slowed down after the negative outcome of the initial clinical trials with trastuzumab plus chemotherapy in patients with HER-2-immunohistochemistry positive NSCLC [27,28].

HER-2 protein overexpression are reported in 2-9% (IHC 3+) [29-32] and 20% (IHC 2+) [29-31] and gene amplification are reported in 2-20% of NSCLC [30-32]. Moreover, HER-2 amplification was present in 13% of the cases at the time of resistance to EGFR TKI [11]. The role of HER-2 overexpression and amplification in lung cancer remains controversial. In a meta-analysis of 40 studies in NSCLC, HER-2 overexpression assessed by IHC was associated with poor prognosis, specifically in adenocarcinomas [33]. Conversely, HER-2 amplification determined by FISH had no prognostic role [33]. HER-2 mutations are present in about 2-4% of NSCLC, especially in women, never-smokers, Asian patients and in adenocarcinomas without EGFR or K-RAS mutations [34-38]. In a population of EGFR/K-RAS/ALK-mutation negative patients, HER-2 mutations can reach up to 6% [34].

T-DM1 has been extensively studied in preclinical models of breast cancer [15,18] These studies demonstrated that T-DM1 has dual mechanisms of action: selective delivery of DM1 to the HER-2-positive tumor cells and activation of antibody-dependent cellular cytotoxicity. T-DM1 demonstrated activity in both trastuzumab and lapatinib resistant HER-2 positive cancer models [18]. T-DM1 was effective also in gastric cell lines [22] and antiproliferative properties have been reported in ovarian SK-OV-3 cell line and in the NSCLC Calu-3 cell line and xenografts [39].

In a recent review Landi and Cappuzzo [40] hypothesized that T-DM1 could play an important role even in NSCLC, and underlined the need of a proper investigation of the real impact of T-DM1 in lung cancer.

All the above observations prompted us to investigate, in a panel of NSCLC cell lines with different levels of HER-2 expression or carrying HER-2 mutation, the effect of T-DM1 on cell proliferation and survival.

In agreement with previously reported data in breast cancer models [15], we documented that also in NSCLC cell lines T-DM1 efficiently inhibited proliferation with arrest in G2-M phase. Moreover, T-DM1 induced cell death by apoptosis in cells with a significant level of surface expression of HER-2 while cells with low level of HER-2 failed to respond to the drug. Interestingly, trastuzumab did not inhibit cell proliferation irrespective of HER-2 expression. H1781 cell line, harbouring mutated HER-2 (G776insV_G/C), was also included in our study. The effect of T-DM1 in this cell line was presumably related to HER-2 level and not affected by the presence of the mutation. Antibody-dependent cytotoxicity assay performed with NK cells demonstrated that T-DM1 retained the activity of trastuzumab as previously reported in breast and gastric models [18,22]. Moreover, we demonstrated that T-DM1 is able to inhibit the growth of a EGFR mutant cell line in which HER-2 overexpression confers resistance to gefitinib. Therefore, targeting HER-2 with T-DM1 might represent a potential approach to overcome EFGR-TKI resistance.

Our in vitro and in vivo experiments documented that, respectively, low cell density and small xenografted tumors were associated with higher HER-2 expression and thereby greater T-DM1 sensitivity. Thus, the present investigation strongly support the contention that HER-2 expression in NSCLC is regulated by the tumor mass and its structural organization which in turn condition the efficacy of T-DM1. Finally, we suggest that the expression level of HER-2, determined by immunohistochemistry, might represent a predictive factor of response to T-DM1 in tumors carrying wild type or mutant HER-2 receptor.

## Conclusions

Our results indicate that T-DM1 inhibited cell proliferation and induced apoptosis in NSCLC cells with endogenous or acquired high HER-2 levels but its activity does not seem to be related to HER-2 mutational status. Moreover, a correlation between cell density/tumour size with both HER-2 expression and T-DM1 activity was established in vitro and in an in vivo xenograft model.

In conclusion, dual-agent molecular targeting through T-DM1 may be a promising therapy in HER-2 positive lung cancer even in tumors which had developed resistance to EGFR-TKIs.

## Methods

### Cell culture

The human NSCLC cell lines used in this study were purchased from American Type Culture Collection (ATCC) (Manassas, VA, USA) and banked at early passage (P2). Furthermore, the cells we culture, are regularly verified on the basis of cell morphology and never cultured for more than 3 months. The PC9, HCC827, HCC827 GR5 and H1781 cell lines were kindly provided in 2013 by Dr P. Jänne (Dana-Farber Cancer Institute, Boston MA, USA). The PC9/HER2c1 was kindly provided in 2013 by Dr.William Pao (Vanderbilt-Ingram Cancer Center, Nashville, Tennessee). All cells were cultured as recommended and maintained at 37°C in a humidified atmosphere of 5% CO_2_ and 95% air.

### Drugs

Trastuzumab and vinorelbine were kindly provided by inpatient pharmacy. T-DM1 was supplied from Genentech Inc. (South San Francisco, CA) through a Materials Transfer Agreement. For the in vitro experiments, stock solutions of drugs were prepared in distilled water, stored at 4°C and diluted in fresh medium for use, whereas for the in vivo experiments trastuzumab and T-DM1 were daily dissolved in sterile saline solution (NaCl 0.9%).

### Western immunoblot analysis

Cell protein extraction, solubilization, and analysis by 1-D PAGE were performed as previously described [41]. Antibodies against HER2; p-HER2 ^Tyr1221/1222^; p70S6K; p-p70S6K ^Thr421/Ser424^; Akt; p-Akt ^Ser473^; p44/42 MAPK; p-p44/42 MAPK; caspase-7 and 9; cyclin A and B1; Rb; p-Rb were from Cell Signaling Technology (Beverly, MA). Antibody against cytochrome-*c* (7H8) was form Santa Cruz Biotechnology Inc. (Dallas, TX). Antibodies against actin was from Sigma–Aldrich (St Louis, MO). Antibody against GAPDH was from Ambion (Austin, TX). HRP-conjugated secondary antibodies were from Pierce (Rockford, IL) and chemiluminescence system (ImmobilionTM Western Cemiluminescent HRP Substrate), was from Millipore (Temecula, CA).

### Flow cytometry

One million of NSCLC cell lines were incubated, for one hour at room temperature, with Isotype control Monoclonal Mouse IgG1/R-PE (Ancell IRP, Bayport, MN, USA) or PE mouse anti-Human HER-2 (BD Biosciences, San Josè CA) to determine HER-2 protein membrane levels as previously described [42]. After the incubation the analysis was performed using an EPICS-XL flow cytometer. Mean fluorescence intensity (MFI) values were converted in units of equivalent fluorochrome (MEF) using the FluoroSpheres 6-Peak Kit (Dako, CA, USA).

### Analysis of cell proliferation and cell cycle

Cell viability was evaluated by tetrazolium dye [3-(4,5-dimethylthiazol-2-yl)-2,5-diphenyltetrazolium- bromide] (MTT) assay and by crystal violet staining as previously described [43]. Data are expressed as percent inhibition of cell proliferation versus control cells. Distribution of the cells in the cell cycle was determined by PI staining and flow cytometry analysis as described elsewhere [44].

### Detection of apoptosis

Apoptosis was assessed by morphological study: stained (Hoechst 33342, propidium iodide) or unstained cells were observed using light-, phase-contrast- and fluorescence-microscopy. Western blot analysis was performed to evaluate cleavage products of caspase-7 and caspase-9. Cytosolic and mitochondrial fractions for cytochrome-*c* detection were generated using a digitonin-based subcellular fractionation technique as previously described [45].

### Isolation and culture of NK cells and ADCC assay

Highly purified CD56+ natural killer (NK) cells were obtained by magnetic separation and ADCC was measured with the CytoTox 96 non-radioactive cytotoxicity assay (Promega, Madison, WI, USA) as previously described [42].

### Tumor xenografts

All experiments involving animals and their care were performed with the approval of the Local Ethical Committee of the University of Parma, in accordance with the institutional guidelines that are in compliance with national (DL116/92) and international (86/609/CEE) laws and policies.

Balb/c-Nude female mice (Charles River Laboratories, Calco, Italy) 6 weeks old, were housed in a protected unit for immunodeficient animals with 12-hour light/dark cycles and provided with sterilized food and water ad libitum [41,42]. We performed an in vivo experiment in order to investigate whether the tumor dimension could influence drug efficacy. 200 μl of matrigel (BD Biosciences) and sterile PBS (1:1) containing 4 × 10^6^ or 8 × 10^6^ Calu-3 cells were subcutaneously injected on the right flank of each mouse. Ten days after cells injection, tumor volume reached an average size of 160 mm^3^ or 370 mm^3^ respectively, and animals were randomized into three different groups (n = 6): control, trastuzumab and T-DM1. Once every six days trastuzumab was intraperitoneally administered at a dosage of 15 mg/Kg, whereas T-DM1 was injected into the lateral tail vein at a dosage of 15 mg/Kg. Control mice received intravenous injection of sterile saline solution (NaCl 0.9%) according to the same schedule. Drug dosages were chosen accordingly to previous studies conducted on breast and gastric xenograft models [15,17,18,22].

Tumor xenografts were measured three times per week using a digital caliper and tumor volume was determined using the formula: (length × width^2^)/2. At the end of the experiments, mice were euthanized by cervical dislocation and tumors weighted and collected for immunohistochemical and histological analysis.

### Morphometric and immunohistochemical analysis of tumor xenografts

Formalin fixed samples were embedded in paraffin. On each tumor serial sections of 5 μm thickness were stained with Haematoxylin and Eosin (H&E), Masson’s Trichrome and subjected to immunohistochemistry. A morphometric analysis was performed on the entire section in order to evaluate the volume of neoplastic tissue, connective tissue and vascular interstitium. To better define the fraction occupied by neoplastic cells, sections were stained with pancytokeratin antibody (PanCK monoclonal mouse, 1:500, o.n. 4°C, Dako) revealed through biotin-streptavidin-DAB system (Dako). The volume fraction of fibrosis and vascular interstitium was assessed on Masson’s Trichrome stained samples. To this end, the number of points overlying each tissue components was counted and expressed as percentage of the total number of points explored. All these morphometric measurements were obtained with the aid of a grid defining a tissue area of 0.23 mm^2^ and containing 42 sampling points each covering an area of 0.0052 mm^2^.

Combining the entire tumor volume with the above morphometric measurements, the total volume occupied by neoplastic cells, connective tissue and vascular interstitium was computed for each sample.

Moreover, immunohistochemical analysis of HER-2 was performed on each tumor of each experimental group. Sections were stained with HER-2 antibody (monoclonal mouse clone 4B5, Ventana, USA) and revealed through biotin-streptavidin-DAB system. This analysis was performed on the entire tumor by an optical microscope (Olympus, BX60- 100X magnification) to evaluate the area occupied by cell expressing HER-2 and their intensity. The latter was expressed as Integrated Optical density (IOD), as detected using a software for image analysis (Image Pro Plus, Media Cybernetics, USA).

In addition, the nuclear expression of the phosphorylated form of Histone H3 (pH-H3, rabbit poyclonal, 1:100, Millipore, USA) on Pancytokeratin^pos^ cells was detected by immunofluorescence to document mitotic figures.

For all tested epitopes negative controls were represented by immunostaining the sample with an irrelevant antibody or by exposing the sections only to the secondary antibody.

### Statistical analysis

Statistical analyses were carried out using GraphPad Prism version 5.0 software (GraphPad Software Inc., San Diego, CA, USA). Results are expressed as mean values ± standard deviations (SD) for the indicated number of independent measurements. Differences between the mean values recorded for different experimental conditions were evaluated by Student’s *t* test, and P values are indicated where appropriate in the figures and in their legends. A P value <0.05 was considered as significant. Bliss interaction was calculated as previously described [46]. For in vivo studies comparison among groups was made using analysis of variance (two-way ANOVA repeated measures) followed by Bonferroni’s post-test.

## Abbreviations

ADCC: Antibody-dependent cellular-cytotoxicity; EGFR: Epidermal growth factor receptor; NSCLC: Non small cell lung cancer; TKI: Tyrosine kinase inhibitor.

## Competing interests

All authors declare that they have no competing interests.

## Authors’ contribution

DC carried out flow cytometry, cell cycle, experiments on resistant clone and analyzed the results; FS performed the in vivo studies, interpreted the results and performed the statistical analysis; FQ, CF and GG carried out morphometric and immunohistochemical analysis; MB carried out Western blot analysis; CF evaluated cell death; AC isolated and cultured NK cells and carried out ADCC experiments; SLM, MG and CC carried out cell growth experiments; LA was responsible for drug administration in in vivo studies; MT, AA and PGP critically revised the manuscript and assisted with the draft of the manuscript; RRA, designed the project, analyzed the results and wrote the manuscript. All authors read and approved the final manuscript.
